# Thyroid hormones regulate cardiac repolarization and QT-interval related gene expression in hiPSC cardiomyocytes

**DOI:** 10.1038/s41598-021-04659-w

**Published:** 2022-01-12

**Authors:** Alessandra Ulivieri, Luca Lavra, Fiorenza Magi, Alessandra Morgante, Leonardo Calò, Patrizio Polisca, Leila B. Salehi, Salvatore Sciacchitano

**Affiliations:** 1 Laboratory of Biomedical Research, Niccolò Cusano University Foundation, Via Don Carlo Gnocchi 3, 00166 Rome, Italy; 2 Department of Cardiology, Policlinico Casilino, Via Casilina, 1049, 00169 Rome, Italy; 3 Medical Genetics Units, PTV, Tor Vergata University Hospital, Via Montpellier, 1, 00133 Rome, Italy; 4 Department of Clinical and Molecular Medicine, Sapienza University, Via di Grottarossa 1035-1039, 00189 Rome, Italy

**Keywords:** Cell biology, Stem cells, Cardiology, Endocrinology

## Abstract

Prolongation of cardiac repolarization (QT interval) represents a dangerous and potentially life-threatening electrical event affecting the heart. Thyroid hormones (THs) are critical for cardiac development and heart function. However, little is known about THs influence on ventricular repolarization and controversial effects on QT prolongation are reported. Human iPSC-derived cardiomyocytes (hiPSC-CMs) and multielectrode array (MEA) systems were used to investigate the influence of 3,3′,5-triiodo-l-Thyronine (T3) and 3,3′,5,5′-tetraiodo-l-Thyronine (T4) on corrected Field Potential Duration (FPDc), the in vitro analog of QT interval, and on local extracellular Action Potential Duration (APD). Treatment with high THs doses induces a significant prolongation of both FPDc and APD, with the strongest increase reached after 24 h exposure. Preincubation with reverse T3 (rT3), a specific antagonist for nuclear TH receptor binding, significantly reduces T3 effects on FPDc, suggesting a TRs-mediated transcriptional mechanism. RNA-seq analysis showed significant deregulation in genes involved in cardiac repolarization pathways, including several QT-interval related genes. In conclusion, long-time administration of high THs doses induces FPDc prolongation in hiPSC-CMs probably through the modulation of genes linked to QT-interval regulation. These results open the way to investigate new potential diagnostic biomarkers and specific targeted therapies for cardiac repolarization dysfunctions.

## Introduction

The delayed ventricular repolarization (QT prolongation) is a dangerous event for the heart. Many studies have emphasized the role of even small perturbations in this process in triggering malignant and often lethal arrhythmias^1,2^. The heart represents one of the major target organs of thyroid function in humans^3,4^ and thyroid dysfunctions, in the forms of overt and subclinical presentations, has been associated with increased cardiovascular mortality as well as morbidity^5,6^. In addition, an altered thyroid function was associated with changes in several important ECG parameters^7^. Hyperthyroidism is known to be an important factor in the etiology of atria and ventricles arrhythmias^3,4^. However, conflicting results have been reported in the literature regarding the role of THs dysfunctions on changes in ventricular repolarization. Both hyperthyroidism and hypothyroidism, in fact, have been reported to be responsible for changes in QT interval duration, with hypothyroidism associated with a prolongation of the QTc interval and hyperthyroidism associated with both decreased and increased repolarization times^8–19^.

A better knowledge on the THs effects on the ventricular repolarization could be relevant in particular for patients affected by cardiac channelopathies, known as Long QT (LQTS) as well as Short QT (SQTS) syndromes, in which abnormal QT interval is often responsible for the sudden cardiac death (SCD) both in newborns and in young adults^20,21^.

The human induced pluripotent stem cell-derived cardiomyocytes (hiPSC-CMs) represent an excellent model to analyze, directly at the single cell level, specific compound cardiotoxicity. This model gives also the possibility to study the hormonal effects on QT interval duration directly in a human context and in a controlled environment^22,23^. In particular, the measurement of FPD by Multi Electrode Arrays (MEA) systems in hiPSC-CMs has been demonstrated to correlate with cardiac action potential as well as to QT interval in ECG in vivo^24–26^.

Aim of this study was to characterize the direct effects of THs on the repolarization of hiPSC-CMs by MEA analysis. Our results demonstrated that T3 and T4 at high doses prolong FPDc of hiPSC-CM through the deregulation of genes linked with cardiac electrophysiological pathways, including QT-interval related genes. The deep characterization of these THs-responsive genes could help to better understand the mechanisms involved in cardiac repolarization and provides new diagnostic and potential therapeutic tools for patients affected by cardiac diseases due to QT interval alterations.

## Results

### The quality of hiPSC-CMs

**Figure 1:**
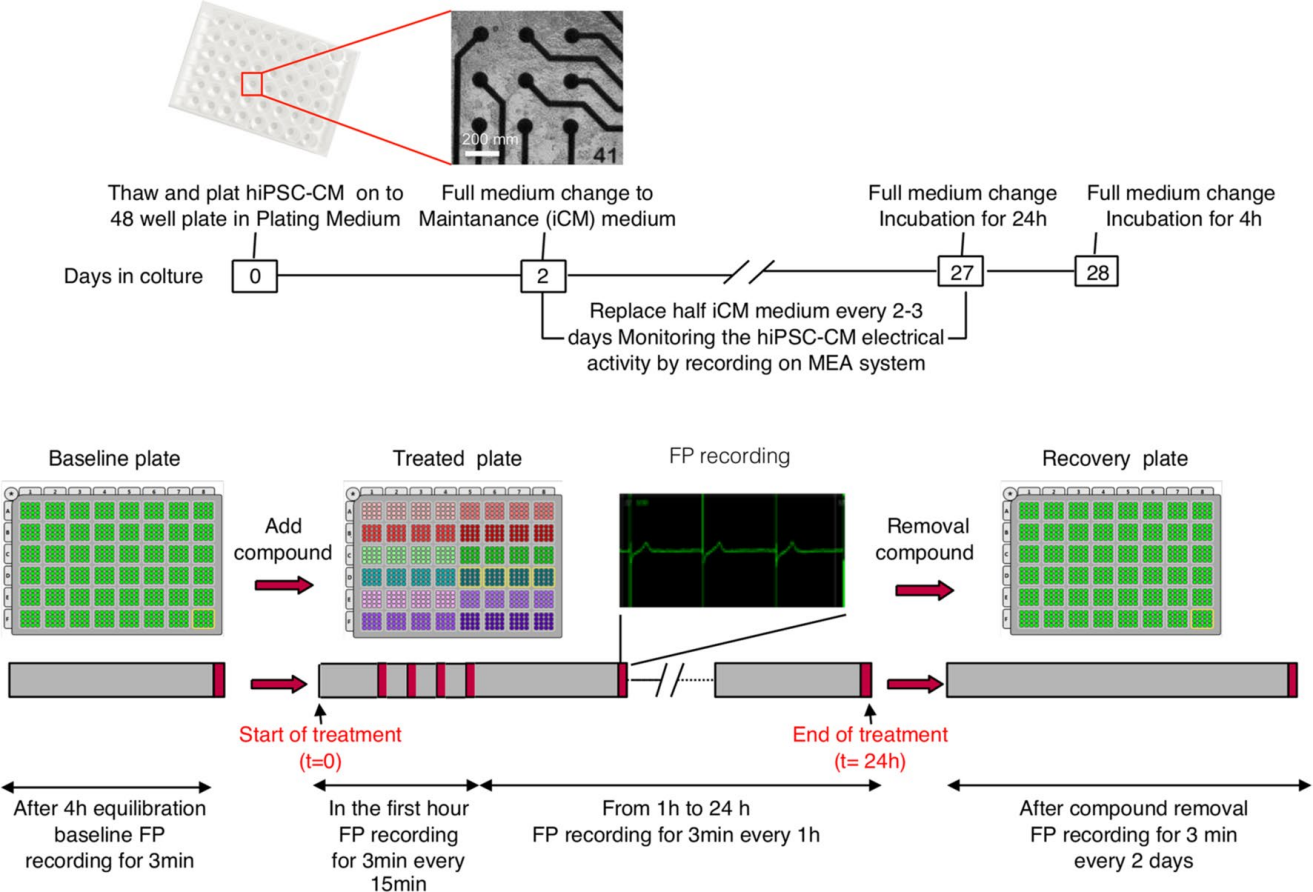
Schematic representation of MEA experimental timeline. **(a**) scheme of MEA probe plating and maturation protocol of hiPSC-CMs. (**b**) schematic illustration of treatments and field potential (FP) MEA recordings time (red box). For a detailed description see “MEA experimental protocol”.

The quality of hiPSC-CMs was evaluated for the plating efficiency, beating rate variability, MEA spike amplitude and drug-response in iCM medium using MEA analysis. The scheme of preparation and subsequent treatment of hiPSC-CMs is reported in Fig. 1. For each lot of cells used in this study, the plating efficiency was > 50%; the coefficient of variation of beating period was < 5%; and the MEA spike amplitude was > 8 mV. Each lot of hiPSC-CMs were tested for their responses to two specific ion channels inhibitors, E-4031 (IKr blocker) and the Nifedipine (L-type Ca^2+^ channel blocker), known to modulate the FPD. The E-4031 administration induced a significant prolongation of FPDc while an evident shortening was observed after Nifedipine treatment (Supplementary Fig. 1a), confirming the previously reported drug-responsiveness of these cells^25^.

### THs induce prolongation of FPDc on hiPSC-CMs

In order to analyze the effects of THs on FPD, hiPSC-CMs plated on MEA probes were treated with different doses of T3 and T4, the biologically active forms of THs.

**Figure 2:**
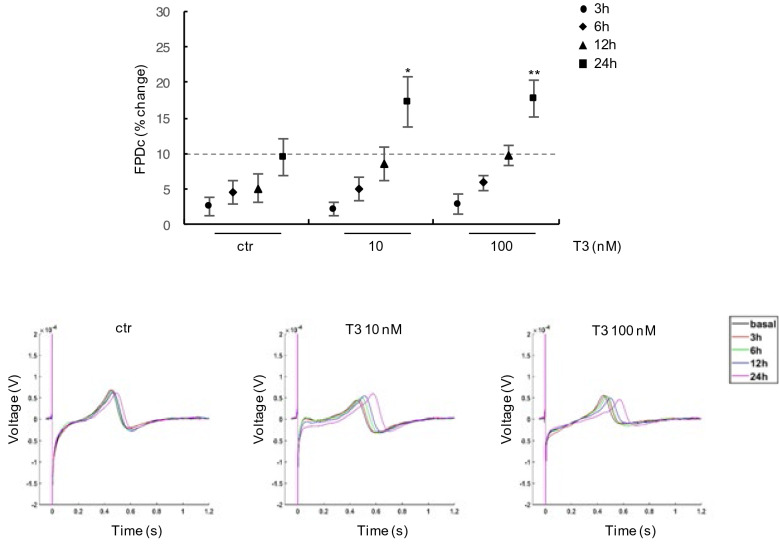
Effect of T3 treatment on FPDc in hiPSC-CMs. Upper panel: analysis of FPDc in hiPSC-CMs treated for 3, 6, 12 and 24 h with 10 nM and 100 nM T3 compared with vehicle treated cells (ctr); data are expressed as the percentage change of FPDc compared to baseline values; lower panel: representative extracted FPD waveforms of hiPSC-CMs treated with T3 at the indicated doses. *p < 0.05 and **p < 0.005.

The cells were initially treated in serum-based medium with T3 doses of 10 nM and 100 nM, concentrations near those reported to induce responses in vitro cardiac cell models^27,28^. Both T3 doses induced a significant prolongation of FPD after 24 h post treatment (% FPDc change: 17 ± 3% T3 10 nM p < 0.05; 17 ± 2.5% T3 100 nM, p < 0.005) and this effect was early observed at 12 h post treatment with the highest T3 dose (% FPDc change T3 100 nM: 10 ± 1%, p < 0.05). No T3 effects on FDPc prolongation were observed at short times (between 15 min to 6 h) of treatment (Supplementary Fig. 2 and Fig. 2).

To analyze the effects of THs on hiPSC-CMs at doses near to physiological levels (FT3 euthyroid range: 4.6 to 9.7 pmol/l, median 6.63 pmol/l.; FT4 euthyroid range: 15.67 and 30.66 pmol/l; median 21.98 pmol/l)^29^, cells were treated with lower hormone concentrations in a serum-free medium for 24 h. The hiPSC-CMs responsiveness to E4031 and Nifedipine in this medium was confirmed (Supplementary Fig. 1b).

**Figure 3:**
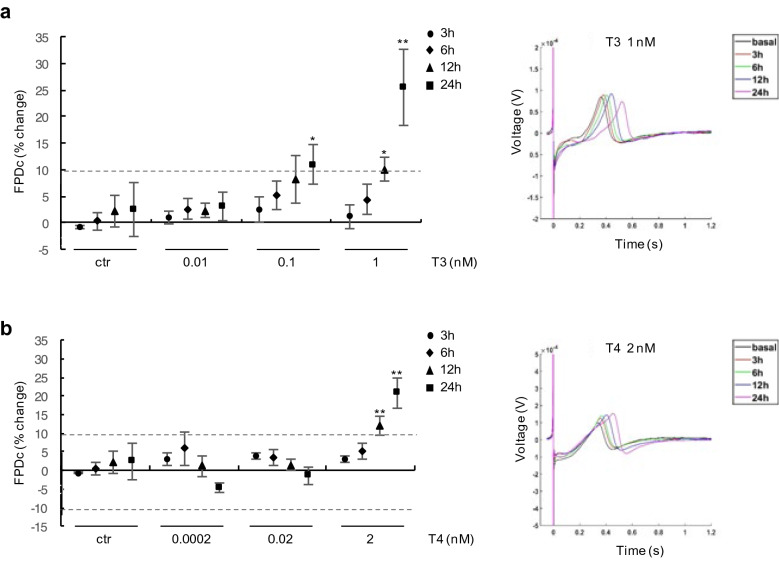
Effect of physiological doses of THs on FPDc in hiPSC-CMs. (**a**) left panel: analysis of FPDc in hiPSC-CMs treated for 3, 6, 12 and 24 h with T3 at different doses (0.01 nM, 0.1 nM, 1 nM) compared with vehicle treated cells (ctr); data are expressed as the percentage change of FPDc compared to baseline values; right panel: representative extracted FPD waveforms of hiPSC-CMs treated with 1 nM T3 at the indicated times. (**b**) left panel: analysis of FPD in hiPSC-CMs treated for 3, 6, 12 and 24 h with T4 at different doses (0.0002 nM, 0.02 nM, 2 nM), compared with vehicle treated cells (ctr); data are expressed as the percentage change of FPDc compared to baseline values; right panel: extracted FPD waveforms of hiPSC-CMs treated with 2 nM T4 at the indicated times. *p < 0.05 and **p < 0.005.

The results of analysis performed after 3, 6, 12 and 24 h respectively are reported in Fig. 3. As shown in graph and in waveform, the lowest T3 (0.01 nM) and T4 doses (0.0002 and 0.02 nM) had no effect on FPDc (% FPDc change < 10%), while the highest T3 (1 nM) and T4 (2 nM) doses induced a moderate FPDc increase after 12 h of treatment (% FPDc change: 10 ± 2% T3 1 nM p < 0.05; 12 ± 2.5% T4 2 nM, p < 0.005) (Fig. 3a,b). After 24 h exposure, a moderate FPDc prolongation was also recorded with 0.1 nM T3 (% FPDc change: 11 ± 4% p < 0.05), but the strongest FPDc prolongation was detected in cells exposed to the highest THs doses (% FPDc change: 25 ± 7% for T3 1 nM and 21 ± 4% for T4 2 nM, p < 0.005) (Fig. 3a,b). These data have been confirmed in two different lots of hiPSC-CMs (Supplementary Fig. 3). No modification of FPDc was observed at short times (between 15 min to 6 h) of THs treatment (Supplementary Fig. 2 and Fig. 3).

These results indicate that THs directly modulate FPDc in hiPSC-CMs and high THs-doses induce FPDc prolongation in these cells.

### T3 induces local extracellular APD prolongation

**Figure 4:**
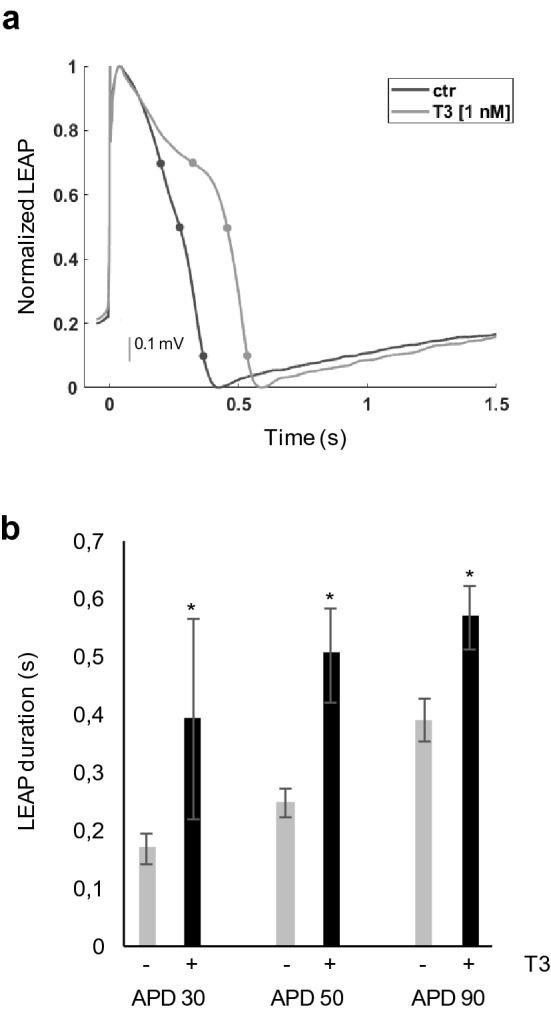
LEAP analysis of hiPSC-CM treated with T3. (**a**) Local extracellular APD analysis of iPSC-CM treated for 24 h with T3 1 nM. The amplitude normalized LEAP waveforms (averaged across 5 beats) from representative wells are overlaid for the vehicle control (grey) and the T3 1 nM (black) treated cardiomyocytes. (**b**) The histogram represents the mean ± standard deviation of APD30, 50, and 90 measured in four wells. *p < 0.05.

To better characterize the TH-induced response, we also performed LEAP induction in TH-treated hiPSC-CMs. Since the stronger effect on FPD was observed with T3 at 1 nM and after 24 h of treatment, cardiomyocytes were treated with these conditions (Fig. 4). After LEAP induction, a significant prolongation of APD was observed in T3-treated compared with vehicle control cells, with the strongest effect in the early phase of repolarization (% APD change: 130% for APD_30_, 100% for APD_50_ and 45% for APD_90_) (Fig. 4a,b).

The T3-induced APD prolongation was consistent with the effect on FPD observed after 24 h in cells exposed to 1 nM T3 compared with vehicle control cells (% FPD change: 37%).

These results suggest that the long-term TH administration induces FPD prolongation probably through an effect on the early phases of the AP repolarization.

### T3 effects on FPDc are mediated by interaction with thyroid hormone nuclear receptors

**Figure 5:**
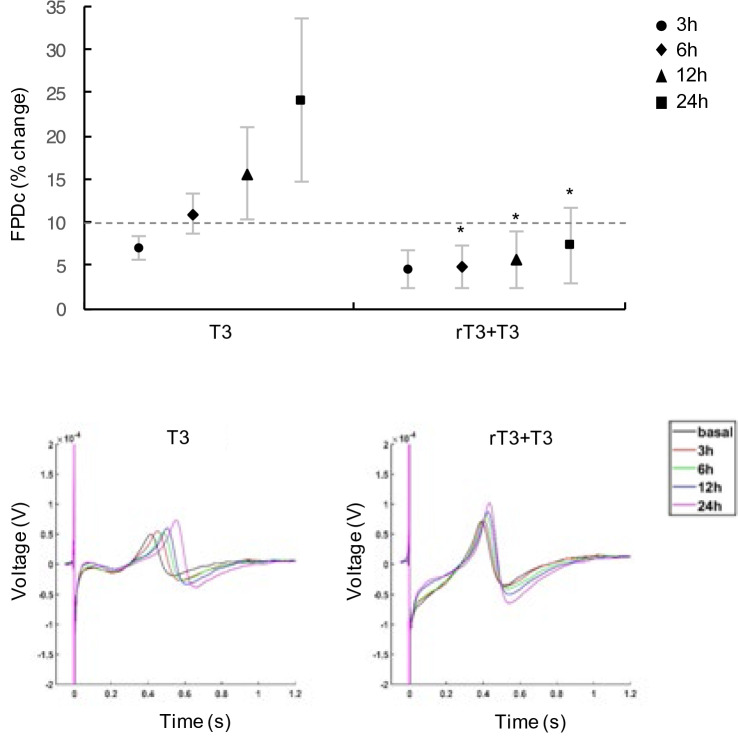
Reverse T3 blocks the T3-dependent FPDc prolongation. Analysis of FPDc T3-treated cells after the preincubation with 1 nM rT3 or rT3 vehicle (ammonia solution) at the indicated times. Data are expressed as the percentage change of FPDc compared to baseline values; lower panel: representative extracted FPD waveforms of hiPSC-CMs treated as previously described. *p < 0.05.

T3 primarily exerts its effects by binding to thyroid hormone nuclear receptors (TRs) on thyroid hormone responsive elements (TRE)s, located in the promoter region of target genes, thus affecting gene expression^30^. However, T3 is also able to exert its effects via another receptor-independent pathway, which largely occurs at the plasma membrane, and involves the regulation of ion transporters activities^31^. To test whether the T3-induced FPDc prolongation could be due to a TR-specific transcriptional gene target modulation or a non-genomic action of TH, before T3 treatment the cells were preincubated with rT3. This is a specific T3 antagonist unable to induce transcriptional effects but able to bind the TRs blocking the T3-mediated transcriptional activity on specific target genes^28,32^ and inhibiting the type 1 model of action, according to the recently proposed new classification of thyroid hormone signaling pathway^33^. As shown in Fig. 5, after 24 h of 1 nM T3 exposure, a significant FPDc prolongation was confirmed in hiPSC-CMs preincubated with the vehicle control (ammonia solution + T3) (% FPDc change: 24 ± 9%, p < 0.05) while no FPDc increase was recorded in T3-treated cells after the preincubation with 1 nM rT3 (% FPDc change: 7 ± 4%). No prolongation of FPDc was observed in cells treated with rT3 alone compared with vehicle control (ammonia solution) treated cells (Supplementary Fig. 4).

The rT3 block of the T3-mediated FPDc prolongation suggests that THs effect is mediated by a TR-specific gene target modulation.

### T3-induced changes in the expression of genes linked to QT-interval regulation

**Figure 6:**
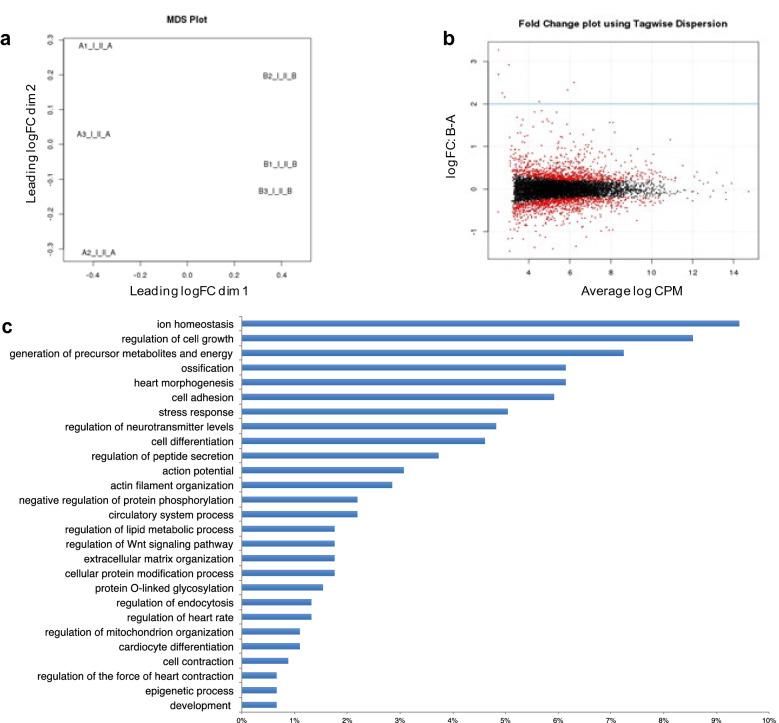
RNAseq analysis identifies T3-induced gene expression modifications in hiPSC-CMs. (**a**) Multidimensional Scaling (MDS) plot showed a clear separation between three replicates of control (A1-A3) and T3-treated (B1-B3) hiPSC-CMs samples. (**b**) Smear Plot of RNA-seq data of different gene expression profile between two groups. Average signal intensity (log CPM versus logFC) of DEGs between ctr and T3 treated iPSC-CMs are shown. The FDR cut-off 0.05 was applied to display the significant DEGs, which are highlighted in red, while non-significant changes are shown in black. Blue horizontal lines represent log2FC. (**c**) GO.BP functional enrichment results bar plot. Length of bar indicates the percentage of genes in each biological process.

To gain additional insight into the molecular mechanisms involved in T3-mediated FPDc prolongation, RNA-seq analysis was performed in hiPSC-CMs after 1 nM T3 treatment. The differential expression analysis of RNA-seq data showed a clear separation between the three replicates of control (A1–A3) and T3-treated (B1-B3) hiPSC-CMs samples (Fig. 6a) and a different gene expression profile between the two groups (Fig. 6b) with a total of 1631 differentially expressed genes (DEGs) (GSE172348_Differential_Expression_AvsB.xlsx). Among these, 736 were significantly deregulated after 24 h exposure to T3 1 nM (FDR < 0.05), with 337 down-regulated and 399 up-regulated genes. (GSE172348_Differential_Expression_AvsB.xlsx).

To identify the biological pathways associated with DEGs altered after T3 treatment, a functional enrichment analysis was performed. GO.BP database analysis identified 634 pathways associated with the 736 DEGs (GSE172348_Functional_Analysis_AvsB.xlsx). Among these, the 10 more significant pathways (p < 3 × 10^–7^) are enriched for a common set of genes related to cardiac functions. Moreover, a further functional GO.BP enriched analysis, filtering data for specific cardiac terms, showed that 62% of DEGs (456/736) are implicated in cardiac biological processes, with the highest percentage of them (9.4%) associated with the ion homeostasis pathway that, together with the action potential pathway (3,1%), are directly involved in cardiac repolarization (Fig. 6c)^34^. Different other pathways identified with GO.BP enriched analysis, spanning from cardiac structure associated functions (actin filament, cell adhesion and extracellular matrix organization) to those linked to protein trafficking (regulation of peptide secretion and endocytosis), protein modification (phosphorylation, O-glycosylation, peptide secretion), cellular metabolism and energy (mitochondrial organization, stress response) in addition to other biological pathways (ossification, cell growth, endocytosis, epigenetic modification) (Fig. 6c), are also associated with cardiac dysfunctions^35–40^.

Interestingly, several TH-induced DEGs identified in our study have been previously related to QT-interval regulation (Table 1)^41–58^.

**Table 1:** T3-induced DEGs associated with QT-interval regulation.

Gene symbol	Gene ID	Gene description	Biological process	Ref
ABCC1	4363	ATP binding cassette subfamily C member 1	Ion homeostasis organic anion transport	^41,42^
ATP1A1	476	ATPase Na^+^/K^+^ transporting subunit alpha 1	Ion homeostasis and action potential	^43^
ATP1B1	481	ATPase Na^+^/K^+^ transporting subunit beta 1	Ion homeostasis and action potential	^45,46^
ATP2A2^a^	488	ATPase sarcoplasmic/endoplasmic reticulum Ca^2+^ transporting 2	Ion homeostasis calcium ion transport	^4,41,46,55^
CACNA2D2	9254	Calcium voltage-gated channel auxiliary subunit alpha2delta 2	Ion homeostasis calcium ion transport	^51^
CACNB2	783	Calcium voltage-gated channel auxiliary subunit beta 2	Ion homeostasis calcium ion transport	^50,51^
HCN4^a^	10021	Hyperpolarization activated cyclic nucleotide gated potassium channel 4	Ion homeostasis and action potential	^4,41,49,55^
KCNJ5	3762	Potassium inwardly rectifying channel subfamily J member 5	Ion homeostasis potassium ion transport	^44^
NEDD4L	23327	NEDD4 like E3 ubiquitin protein ligase	Ion homeostasis	^47^
SLMAP	7871	Sarcolemma associated protein	Action potential	^48^
SNTA1	6640	Syntrophin alpha 1	Action potential	^46^
DNAJB11	51726	DnaJ heat shock protein family (Hsp40) member B11	Involved in protein folding	^58^
DRD1	1812	Dopamine receptor D1	Regulation of neurotransmitter levels	^41,42,51^
HSPA8	3312	Heat shock protein family A (Hsp70) member 8	Involved in protein folding	^58^
KDR	3791	Kinase insert domain receptor	Cell adhesion regulation of cell–matrix adhesion	^42^
MKL2	416421	Myocardin related transcription factor B	Muscle organ development	^46^
MPP2^a^	4355	Membrane palmitoylated protein 2	Excitatory postsynaptic potential	^41,57^
MYH6^a^	4624	Myosin heavy chain 6	Cardiac muscle contraction	^4,41,52,55^
NDRG4	65009	NDRG family member 4	Heart morphogenesis	^45^
NOS1AP	9722	Nitric oxide synthase 1 adaptor protein	Regulation of heart rate by chemical signal	^45^
NPPA	4878	Natriuretic peptide A	Cardiac conduction system development	^41,53^
NPPB^a^	4879	Natriuretic peptide B	Cardiac conduction system development	^41,54,56^
SRL	6345	Sarcalumenin GTPase activity	Endosomal transport	^46^
TCEA3	6920	Transcription elongation factor A3	Transcription, DNA-templated	^56^

List of known QT interval-related DEGs identified with RNAseq analysis. Gene nomenclature details were obtained from Entrez Gene database.

^a^Known T3-reponsive genes.

Among them, there are genes directly linked to ion homeostasis and action potential regulation pathways (ABCC1, ABCD1 ATP1A1, ATP2A2, ATP1B1, CACNA2D2, CACNB2, HCN4, KCNJ5, NEDD4L, NOS1AP, SLMAP, SNTA1), together with genes associated with other biological functions (DNAJB11, DRD1, HSPA8, MKL2, MMP2, MYH6, NDRG4, NPPA, NPPB, KDR, SRL, TCEA3) (Table 1). In addition, among these DEGs, some are known myocardial T3-responsive genes (ATP2A2, HCN4, MMP2, MYH6, and NPPB) associated with cardiac repolarization alterations (Table 1)^46,49,52,54–57^.

Altogether these results suggest that T3 treatment of iPSC-CMs at high doses is associated with the modulation of the expression of specific genes implicated in cardiac repolarization and highlight the role of T3 as a master regulator of cardiomyocyte electrical activity at different levels.

## Discussion

Clinicians (cardiologists as well as endocrinologists) are well aware that when they face a patient affected by thyroid dysfunctions his/her heart may suffer potential negative effects of the altered thyroid status as well as of an inappropriate treatment. Sinus tachycardia, atrial fibrillation and arrhythmias are frequently observed in patients with thyroid dysfunctions and are considered hallmarks of the hyperthyroid state^3,4^. The effects of THs on QT duration are indeed not so clear and conflicting results have been reported in the literature. In fact, if in the patients with hypothyroidism the QT prolongation and a heightened risk for *torsades de pointes* are well documented^8,9,11^, the effects of hyperthyroidism on ventricular repolarization are more controversial, since both prolonged and shortened QTc intervals have been reported^10,12–19^. Moreover, in a recent large population study, involving more than 130,000 subjects from a Danish National Register^7^, a U-type curve is described in male subjects with QTc prolongation seen in both hyperthyroidism and hypothyroidism, suggesting that maintenance of thyroid homeostasis is necessary to keep the QT interval in the normal range.

Several experimental models have been proposed in the past to study the mechanism of APD prolongation by THs, including primates, dogs, goats, pigs, rabbits, guinea pigs, chickens, rats and mice^59–62^. However, besides the difficulties in translating the results obtained in animals to humans^63^, these cell models are difficult to be maintained in culture for a long period of time. Moreover, differences in ion channel distribution have been demonstrated among species and some of them are not completely understood yet. This makes the extrapolations of experimental findings to humans more complicated^64^. The hiPSC-CMs represent an attractive in vitro model to study cardiomyocyte electrophysiology. They are readily available human-derived cells and can be maintained in vitro for a long time to analyze acute and chronic effects of several compounds (drugs, hormones, etc.). They show a spontaneous stably beating activity and, for this reason, they represent an excellent experimental platform to model cardiovascular diseases. They are currently used for preclinical testing of drug-induced cardiotoxicity, development of new diagnostic as well as therapeutic strategies, and provide an attractive tool for patient-specific disease model to analyze specific mutation in genes coding for ion channels involved in ventricular repolarization, including patients with long QT syndrome^24,65^.

In this study, we used hiPSC-CMs to investigate the direct effect of long time THs treatment on FPD through MEA analysis. These experimental conditions, together with the use of a serum free medium, allowed us to exclude any external effect as well as those exerted in vivo by vessels, nerves, other hormones or by other components of the cardiac muscle tissue^22,23^.

Most of the in vitro studies published so far in the literature concerning the effects of THs on hiPSC-CMs have been focused on their role in driving cardiomyocyte maturation^66^. This cell model, in the early stages of culture, exhibits functional characteristics resembling a fetal rather than an adult cardiomyocyte^67,68^. Such relatively immature phenotypes of the hiPSC-CMs can be corrected by prolonged culture. In fact, it has been recently demonstrated that iCell cardiomyocytes acquire a mature phenotype four weeks after plating^69^ and for this reason we decided to culture cardiomyocytes on MEA probes for up to 28 days before performing our treatment experiments (Fig. 1).

To the best of our knowledge this is the first study aimed to analyze the effects of THs on the electrophysiological activities of hiPSC-CMs. Our results indicate that both T3 and T4 are involved in cardiomyocyte FPDc modulation with a significant FPDc prolongation observed in hiPSC-CMs after 12–24 h of high dose THs exposure (Figs. 2 and 3). The stronger effect on FPDc was detected at 1 nM and 2 nM T3 and T4 concentrations, respectively (Fig. 3a,b). No further increase of FPDc with higher T3 dose was observed, suggesting a putative saturation point reached at 1 nM treatment (Figs. 2, 3). The demonstrated TH-induced FPDc prolongation is consistent with APD increase observed by LEAP analysis after 24 h in cells exposed to 1 nM T3 (Fig. 4). The maximum effect on APD_30_ suggests that THs are probably involved in the early phases of the AP repolarization, where voltage-dependent K^+^ outward currents are responsible for APD, especially in ventricular myocytes^35^.

The THs-induced FPDc prolongation was observed only long time THs administration while no FPDc variation was observed after a short time hormone exposure (Figs. 2, 3 and Supplementary Fig. 2). The timing of the THs effect on FPDc suggests that the mechanism by which THs acts is probably mediated by the TR-specific transcriptional gene target modulation^30^ instead of a rapid non-genomic action (i.e., direct effect on ion channels)^31,34^. The preincubation with the biologically inactive metabolite rT3, that bind the TRs without inducing any transcriptional action^28,32^, is able to block the T3-mediated induction of FPDc (Fig. 5), suggesting that this effect is specific and mediated by the activity of these transcription factors. Both TRs genes (*THRA* and *THRB*) are expressed in our hiPSC-CMs with the TRα mRNA levels higher than TRβ mRNA levels (GSE172348_Filtered_Normalized_Data_AvsB.xlsx and Supplementary Fig. 5), in agreement with the main expression of the TRα reported in heart muscle^70,71^. Based on these evidences we suppose that the regulation of cardiac repolarization in iPSC-CMs is likely due to canonical T3 transcription mechanism (type 1)^42^ probably through the TR alpha. However, a more in-depth investigation at the molecular level will be needed to confirm such hypothesis and to identify the specific TR (α, β or both) involved.

Gene expression profiling analysis (RNAseq analysis), performed on T3-treated hiPSC-CMs, shows a significant dysregulation of 736 genes. Interestingly, most of these are new potential TH-responsive transcripts, not previously described. In particular, a common set of genes related to cardiac electrophysiological activity, mainly belonging to the ion homeostasis, are deregulated after T3-treatment (Fig. 6c). A fine regulation of this process is crucial for the control of cardiac APD due to the sequential activation and inactivation of ion channels carrying inward (Na^+^ and Ca^2+^) and outward (K^+^) currents^34^. In particular, the inhibition of K^+^ currents as well as abnormal Ca^2+^ handling and Na^+^ currents are responsible for a delay repolarization, a prolongation of the QT interval and an increase in the risk of *torsades de pointes,* as often reported in inherited or acquired disease LQTS and SQTS^34,72,73^. Interestingly, in agreement with the ADP/FPDc alteration observed in our iPSC-CMs, several TH-induced DEGs identified in our study are directly associated with these ion currents genes, and a subset of them was also previously related with QT-interval regulation (Table 1).

In addition to cardiac conduction pathways, a subgroup of DEGs identified are associated with other different cardiac biological processes (Fig. 6c) and have been previously linked with QT-interval regulation (Table 1), in agreement with the complexity of the cardiac electrophysiological processes^74^. Most of these genes/processes are known to be regulated by THs^75^ and some of them have also been associated with cardiac conduction dysfunctions^3,43,48,50–54,72–74^, like the known myocardial T3-induced genes linked with QT-interval alteration (*ATP2A2*, *HCN4*, *MMP2*, *MYH6*, and *NPPB*)^46,49,52,54–57^, confirming the relationship between THs-induced modulation of specific genes and the THs-mediated ADP/FPDc alteration observed.

Interestingly, a group of T3-induced DEGs are involved in the regulation of peptide secretion, endocytosis, post-translational modifications (phosphorilation and glicosilation), and endosomal transport (i.e. the QT-interval related gene *SRL*). All these mechanisms could be implicated in ion channel trafficking, a crucial process for the regulation of ion channel function, and cardiac repolarization^35,58,75,76^. In particular, among these genes, two are involved in the hERG ion channel trafficking (*DNAJB11, HSPA8*)^58,76,77^. The alteration of this process is recognized as an important mechanism in hERG channel dysfunction associated with LQTS2 and QT prolongation due to pharmacological treatments^76–79^.

Altogether, these evidences suggested a multilevel role of THs in the regulation of cardiac repolarization as well as an interesting correlation between THs-mediated ADP/FPDc alteration and ion channel trafficking regulation.

Further studies based on functional analysis of the T3-induced DEGs identified in our study will be performed to in deep investigate the role of these genes in the complex mechanisms involved in the THs-induced cardiac repolarization prolongation observed in iPSC-CMs.

In conclusion, our study indicates for the first time that THs treatment of a highly representative human cell model, the hiPSC-CMs, is responsible for APD and FPDc prolongation and that this effect is likely mediated by TRs-mediated gene modulation. Moreover, our study demonstrates that hiPSC-CMs represent an excellent model to perform deep analysis of the effects of THs and to clarify the molecular mechanism of action of THs on cardiac electrophysiological activity. RNA sequencing analysis reveals that, in specific conditions, THs modulate the expression of specific genes previously associated with QT-interval and cardiac electrical alterations. Further experiments will be performed to clarify the specific role of these genes in the THs-mediated APD/FPDc prolongation and to better investigate the molecular mechanisms involved in the fine and complex regulation of cardiac repolarization by these hormones. Additional and future characterization of the candidate effectors of THs action could provide relevant diagnostic and potential therapeutic tools for the management of patients affected by cardiac as well as thyroid disease associated with QT interval alterations.

## Materials and methods

### Cell culture and plating on MEA probe

Cryopreserved hiPSC-derived iCell Cardiomyocyte, obtained from circulating blood fibroblasts of a Caucasian 18 years old female and reprogrammed by retroviral transduction (Cellular Dynamics International, Inc. Madison, WI, USA, clone 01434, lot 1299716 and lot 1591669) were cultivated following manufacturing instructions.

The cardiomyocytes were thawed in iCell Cardiomyocytes Plating Medium and directly plated onto fibronectin-coated CytoView MEA 48 wells white plates (Axion Biosystem, Atlanta, GA, USA) at 3 × 10^4^ plated viable cells per well, based on lot specific plating efficiency. The hiPSC-CMs were incubated at 37 °C with 5% CO_2_ and allowed to attach for 2 h in moisturized conditions, prior filling each well with 0.3 mL of Plating medium. On day 2 post-plating, the spent medium was replaced with iCell Maintenance (iCM) medium and thereafter 50% of the medium was replaced every 2–3 days, up to cell treatment. Before the experiments, hiPSC-CMs were cultured for four weeks after plating to improve their functional maturation, as previously reported^69^. The scheme of preparation and subsequent treatment of hiPSC-CMs for MEA analysis is reported in Fig. 1a,b.

### Drugs and chemicals

The selective ether-a-go-go-related (hERG) channel blocker E-4031 (ABCAM, Cat. # ab12158, Cambridge, MA, USA), and the L-type Ca^2+^ channel blocker Nifedipine (SIGMA-ALDRICH, Cat. #N7634, St Louis, MO) were respectively prepared in distilled water and DMSO as 5 mM and 10 mM stock solutions and used as calibration drugs. The 3,3′,5,-Triiodo-l-thyronine as 30.7 µM (T3, Liotir) and the 3,3′,5,5′-tetraiodo-l-thyronine as 64 µM (T4, levothyroxine sodium) (IBSA farmaceutici Italia s.r.l., Lodi, Italia) stock solutions were used as test compounds as well as the T3 antagonist 3,3′,5′-Triiodo-l-thyronine Reverse T3 (rT3) (SIGMA-ALDRICH, Cat. # T3787, St Louis, MO), prepared as 15.4 mM stock in 4 M ammonia solution in MetOH.

Before cells treatment, all stock solutions as well as vehicle controls (H_2_0 for E4031, DMSO for Nifedipine, EtOH for T3 and T4, and ammonia solution 4 M in MetOH for rT3, were prepared as 10× working solutions in serum based iCM medium or serum-free BMCC medium (Ncardia, Cat. # Ax-M-BMCC250 Leiden, Netherlands) until added to test wells. The added dose of vehicle controls was equivalent to each respective test compound concentration and no more than 0.1%.

### MEA experimental protocol

The electrical behavior of spontaneously beating hiPSC-CMs monolayers was recorded on the Maestro Pro MEA platform (Axion Biosystem, Atlanta, GA, USA). The day before treatment, the cells were fed with serum based (iCM) or serum-free (BMCC) medium. The specific culture media were completely replaced with fresh media prior to compound addition and after 4 h of stabilization in the MEA system at 5% CO_2_ and at 37 °C, baseline activity of spontaneously beating hiPSC-CMs monolayers was recorded (baseline plate, Fig. 1b). Cells were treated at the same time by adding a 1:10 dilution of each 10× compound working solution in 4 replicate sets. The final compound concentrations used were: 5 and 10 nM for E-4031, 10 and 30 nM for Nifedipine, from 0.01 to 100 nM for T3 and from 0.0002 to 2 nM for T4. For rT3 treatment, after baseline recording, cells were preincubated for 3 h with rT3 at concentration of 1 nM or rT3 vehicle (ammonia solution), and then treated with equimolar doses of T3 for 24 h. Post-dose cells (treated cell plate) electrical activities (FP waveform) were recorded for 3 min at 15 min intervals, for the first hour, and then every 3 h for a total of 24 h using AxIS Navigator, version 2.0.4 (Axion Biosystem, Atlanta, GA, USA). At the end of treatment, a cell recovery in serum based medium (iCM) was performed and FP measurements were recorded for 3 min every two days (Fig. 1b).

The local extracellular action potential (LEAP) induction experiments were performed to measure the Action Potential Duration (APD) and morphology after FPD recording. LEAP signals were induced 24 h after T3 treatment (1 nM) and then recorded for 10 min. In each well only half electrodes were stimulated. LEAP measurements were performed also in control wells treated with T3 vehicle (EtOH 0.001%).

### MEA data analysis

Fridericia’s corrected FPD (FPDc) data analysis was performed on Cardiac Analysis Tool version 2.2.7 and AxIS Metric Plotting Tool version 2.2.5 (Axion Biosystem, Atlanta, GA, USA). The golden channel for FPDc measures was selected by the Cardiac Analysis Tool by identifying, in the recorded signals from each experiment, the region of stable beating and the electrode with the largest repolarization feature in baseline condition^25^. Data were expressed as the percentage of FPDc change from baseline, calculated as mean ± SD (n = 4) for each treatment.

LEAP signal analysis was performed on Cardiac Analysis Tool version 2.2.7 (Axion Biosystem, Atlanta, GA, USA) and AxIS Metric Plotting Tool version 2.2.5^26^. LEAP analysis data were expressed as mean ± SD (n = 4) of APD, measured from beat start to 30%, 50%, and 90% voltage repolarization (APD30, APD50, APD90) in T3 (1 nM) and EtOH (0.001%) treated wells.

### RNA-seq analysis

Spontaneously beating hiPSC-CMs monolayers were treated with vehicle control (EtOH 0.01%) or with 1 nM T3 for 24 h. The treatments were performed in triplicate. RNA was isolated using the total RNA mini kit (Norgen Biotek Corp.) following manufacturer’s instructions and RNA concentration was measured using a NanoDrop ND-1000 spectrophotometer (Thermo Fisher Scientific). Library preparation, RNA-sequencing and bioinformatics analysis were done by the service provider GENOMNIA srl (Bresso, Milano). For each RNA sample (n = 3/group), a library was generated and analyzed with Agilent 2100 Bioanalyzer. The reads were mapped and analyzed using Torrent Suite (version 5.12.1). In particular, the AmpliseqRNA plug-in (version 5.1.01) was used to map the sequencing reads versus the human AmpliseqRNA panel (> 22,000 genes). The read count data from RNA-seq were then normalized and used to perform differential expression analysis using the R EdgeR package (version 3.24.3, http://bioconductor.org). Counts were further filtered to include only more differentially expressed genes (DEGs) using a False Discovery Rate (FDR) < 0.05 and log fold change (logFC) < − 1 and > 1 as thresholds. The Transcriptome Analysis Console (TAC version 4.0.2.15, (https://tools.thermofisher.com/content/sfs/brochures/tac_software_datasheet.pdf) software was applied to calculate sample spatial coordinates. These data were used for Multidimensional Scaling (MDS) and Smear Plot analyses, visualized with matplotlib library Python 3 (version 3.1.2). The replicate samples clearly cluster together, indicating the biological and technical data reproducibility. F-test performed with the same software was used to identify genes that significantly discriminated between the two different groups (p < 0.05). These genes were further filtered to select those with the highest differential expression (log Count Per Million, logCPM ≥ 10). A functional and pathway enrichment analysis of DEGs was performed using R cluster Profiler package (version 3.10.1) on Gene Ontology (GO) (Cellular Component CC, Biological Process BP and Molecular Function MF) pathway databases. Moreover, to identified DEGs associated with cardiac pathways, a further functional GO.BP enriched analysis was performed using the following cardiac terms: “atherosclerosis, cardiomyopathy, cardiac, heart, atrioventricular, ion channel, aorta, cardiocyte, circulatory, long QT, action potential, heart rate, arrhythmia”.

### Statistics

Data are calculated as the mean ± standard error of the mean (SEM) and FPD were expressed as the percentage of FPDc change from baseline. Based on CSHAI study^80^, a cut-off value of 10% FPDc variation was chosen and only changes above such percentage were considered. Statistical analysis was performed by using one-way Anova test for variance analysis of multiple groups of measures and the post hoc t-test for the pairwise comparison of combination of group pairs, in particular vehicle treated control cells vs THs treated cells for each time. The statistical significance was accepted for p < 0.05. Analysis has been performed using KaleidaGraph software (Version 4.5.4, https://www.synergy.com/).

## Supplementary Information

Supplementary Figures.

## Data Availability

RNA-seq data have been deposited in the Gene Expression Omnibus (GEO) dataset with the accession code GSE172348 (https://www.ncbi.nlm.nih.gov/geo/query/acc.cgi?acc=GSE172348, secure reviewer token: sngtkywkbjwxrip).

## References

[1] Zareba W, Moss AJ, le Cessie S (1994). Dispersion of ventricular repolarization and arrhythmic cardiac death in coronary artery disease. Am. J. Cardiol..

[2] Ikeda T (2000). Combined assessment of T-wave alternans and late potentials used to predict arrhythmic events after myocardial infarction. A prospective study. J. Am. Coll. Cardiol..

[3] Kahaly GJ, Dillmann WH (2005). Thyroid hormone action in the heart. Endocr. Rev..

[4] Grais IM, Sowers JR (2014). Thyroid and the heart. Am. J. Med..

[5] Bano A (2017). Association of thyroid function with life expectancy with and without cardiovascular disease: The Rotterdam study. JAMA Intern. Med..

[6] Ning Y (2017). What is the association of hypothyroidism with risks of cardiovascular events and mortality? A meta-analysis of 55 cohort studies involving 1,898,314 participants. BMC Med..

[7] Tayal B (2019). Thyroid dysfunction and electrocardiographic changes in subjects without arrhythmias: A cross-sectional study of primary healthcare subjects from Copenhagen. BMJ Open.

[8] Kweon KH, Park BH, Cho CG (2007). The effects of l-thyroxine treatment on QT dispersion in primary hypothyroidism. J. Korean Med. Sci..

[9] Bakiner O (2008). Subclinical hypothyroidism is characterized by increased QT interval dispersion among women. Med. Princ. Pract..

[10] Colzani RM (2001). Hyperthyroidism is associated with lengthening of ventricular repolarization. Clin. Endocrinol..

[11] Galetta F (2008). Changes in heart rate variability and QT dispersion in patients with overt hypothyroidism. Eur. J. Endocrinol..

[12] Dörr M (2006). The relation of thyroid function and ventricular repolarization: Decreased serum thyrotropin levels are associated with short rate-adjusted QT intervals. J. Clin. Endocrinol. Metab..

[13] Gomberg-Maitland M, Frishman WH (1998). Thyroid hormone and cardiovascular disease. Am. Heart J..

[14] Binah O, Arieli R, Beck R, Rosen MR, Palti Y (1987). Ventricular electrophysiological properties: Is interspecies variability related to thyroid state?. Am. J. Physiol..

[15] Owecki M, Michalak A, Nikisch E, Sowiński J (2006). Prolonged ventricular repolarization measured by corrected QT interval (QTc) in subclinical hyperthyroidism. Horm. Metab. Res..

[16] Guntekin U (2009). QTc dispersion in hyperthyroidism and its association with pulmonary hypertension. Pacing Clin. Electrophysiol..

[17] Lee YS (2015). The corrected QT (QTc) prolongation in hyperthyroidism and the association of thyroid hormone with the QTc interval. Korean J. Pediatr..

[18] Van Noord C (2008). High free thyroxine levels are associated with QTc prolongation in males. J. Endocrinol..

[19] Zhang Y (2013). Thyroid hormones and electrocardiographic parameters: Findings from the third national health and nutrition examination survey. PLoS ONE.

[20] Schwartz PJ (1998). Prolongation of the QT interval and the sudden infant death syndrome. N. Engl. J. Med..

[21] O'Neal WT (2017). Association between QT-interval components and sudden cardiac death: The ARIC Study (Atherosclerosis Risk in Communities). Circ. Arrhythm. Electrophysiol..

[22] Shi Y, Inoue H, Wu JC, Yamanaka S (2017). Induced pluripotent stem cell technology: A decade of progress. Nat. Rev. Drug Discov..

[23] Huo J, Wei F, Cai C, Lyn-Cook B, Pang L (2019). Sex-related differences in drug-induced QT prolongation and torsades de pointes: A new model system with human iPSC-CMs. Toxicol. Sci..

[24] Kussauer S, David R, Lemcke H (2019). hiPSCs derived cardiac cells for drug and toxicity screening and disease modeling: What micro-electrode-array analyses can tell us. Cells.

[25] Clements M (2016). Multielectrode array (MEA) assay for profiling electrophysiological drug effects in human stem cell-derived cardiomyocytes. Curr. Protoc. Toxicol..

[26] Hayes HB (2019). Novel method for action potential measurements from intact cardiac monolayers with multiwell microelectrode array technology. Sci. Rep..

[27] Sakaguchi Y, Cui G, Sen L (1996). Acute effects of thyroid hormone on inward rectifier potassium channel currents in guinea pig ventricular myocytes. Endocrinology.

[28] Huang CJ, Geller HM, Green WL, Craelius W (1999). Acute effects of thyroid hormone analogs on sodium currents in neonatal rat myocytes. J. Mol. Cell Cardiol..

[29] Passath A, Leb G, Goebel R (1985). The evaluation of free thyroid hormones (FT4 and FT3) in the routine diagnosis of thyroid function. Nuklearmedizin.

[30] Brent GA (2012). Mechanisms of thyroid hormone action. J. Clin. Investig..

[31] Davis PJ, Goglia F, Leonard JL (2016). Nongenomic actions of thyroid hormone. Nat. Rev. Endocrinol..

[32] Botta JA, de Mendoza D, Morero RD, Farías RN (1983). High affinity l-triiodothyronine binding sites on washed rat erythrocyte membranes. J. Biol. Chem..

[33] Flamant F (2017). Thyroid hormone signaling pathways: Time for a more precise nomenclature. Endocrinology.

[34] Nerbonne JM, Kass RS (2005). Molecular physiology of cardiac repolarization. Physiol. Rev..

[35] Cubeddu LX (2016). Drug-induced inhibition and trafficking disruption of ion channels: Pathogenesis of QT abnormalities and drug-induced fatal arrhythmias. Curr. Cardiol. Rev..

[36] Cerrone M, Delmar M (2014). Desmosomes and the sodium channel complex: Implications for arrhythmogenic cardiomyopathy and Brugada syndrome. Trends Cardiovasc. Med..

[37] Ducheix S, Magré J, Cariou B, Prieur X (2018). Chronic O-GlcNAcylation and diabetic cardiomyopathy: The bitterness of glucose. Front. Endocrinol..

[38] Kou S (2020). Patients with ACVR1R206H mutations have an increased prevalence of cardiac conduction abnormalities on electrocardiogram in a natural history study of Fibrodysplasia Ossificans Progressiva. Orphanet. J. Rare Dis..

[39] Liu M, Liu H, Dudley SC (2010). Reactive oxygen species originating from mitochondria regulate the cardiac sodium channel. Circ. Res..

[40] Papait R, Serio S, Condorelli G (2020). Role of the epigenome in heart failure. Physiol. Rev..

[41] https://maayanlab.cloud/Harmonizome/gene_set/Long+QT+Syndrome/CTD+Gene-Disease+Associations

[42] Mamoshina P, Rodriguez B, Bueno-Orovio A (2021). Toward a broader view of mechanisms of drug cardiotoxicity. Cell Rep. Med..

[43] Pott A (2018). Mutation of the Na+/K+-ATPase Atp1a1a.1 causes QT interval prolongation and bradycardia in zebrafish. J. Mol. Cell Cardiol..

[44] Yang Y (2010). Identification of a Kir3.4 mutation in congenital long QT syndrome. Am. J. Hum. Genet..

[45] Pfeufer A (2009). Common variants at ten loci modulate the QT interval duration in the QTSCD Study. Nat. Genet..

[46] Arking DE (2014). Genetic association study of QT interval highlights role for calcium signaling pathways in myocardial repolarization. Nat. Genet..

[47] Wang Y (2020). Alterations of Nedd4-2-binding capacity in PY-motif of NaV 1.5 channel underlie long QT syndrome and Brugada syndrome. Acta Physiol..

[48] Ishikawa T (2012). A novel disease gene for Brugada syndrome: Sarcolemmal membrane-associated protein gene mutations impair intracellular trafficking of hNav1.5. Circ. Arrhythm. Electrophysiol..

[49] Ueda K, Hirano Y, Higashiuesato Y (2009). Role of HCN4 channel in preventing ventricular arrhythmia. J. Hum. Genet..

[50] Lozano-Velasco E, Aranega A, Franco D (2021). Non-coding RNAs in the cardiac action potential and their impact on arrhythmogenic cardiac diseases. Hearts.

[51] Zhang Q, Chen J, Qin Y, Wang J, Zhou L (2018). Mutations in voltage-gated L-type calcium channel: Implications in cardiac arrhythmia. Channels.

[52] Priest JR, Ceresnak SR, Dewey FE, Malloy-Walton LE, Dunn K, Grove ME, Perez MV, Maeda K, Dubin AM, Ashley EA (2014). Molecular diagnosis of long QT syndrome at 10 days of life by rapid whole genome sequencing. Heart Rhythm.

[53] Qureshi SF (2014). Atrial natriuretic peptide gene—A potential biomarker for long QT syndrome. EXCLI J..

[54] Vrtovec B, Knezevic I, Poglajen G, Sebestjen M, Okrajsek R, Haddad F (2013). Relation of B-type natriuretic peptide level in heart failure to sudden cardiac death in patients with and without QT interval prolongation. Am. J. Cardiol..

[55] Dillmann WH (2002). Cellular action of thyroid hormone on the heart. Thyroid.

[56] Wang K (2020). BNP as a new biomarker of cardiac thyroid hormone function. Front. Physiol..

[57] Ziegelhöffer-Mihalovicová B, Briest W, Baba HA, Rassler B, Zimmer HG (2003). The expression of mRNA of cytokines and of extracellular matrix proteins in triiodothyronine-treated rat hearts. Mol. Cell Biochem..

[58] Smith JL, Anderson CL, Burgess DE, Elayi CS, January CT, Delisle BP (2016). Molecular pathogenesis of long QT syndrome type 2. J. Arrhythm..

[59] Finet JE, Rosenbaum DS, Donahue JK (2009). Information learned from animal models of atrial fibrillation. Cardiol. Clin..

[60] Sharp NA, Neel DS, Parsons RL (1985). Influence of thyroid hormone levels on the electrical and mechanical properties of rabbit papillary muscle. J. Mol. Cell Cardiol..

[61] Johansson C, Koopmann R, Vennström B, Benndorf K (2002). Accelerated inactivation of voltage-dependent K+ outward current in cardiomyocytes from thyroid hormone receptor alpha1-deficient mice. J. Cardiovasc. Electrophysiol..

[62] Shahrara S, Drvota V, Blange I, Törmä H, Sylvén C (1997). Characterization of AT-1 cardiomyocytes as a model for studies of T3 effects on cardiac cells. Biochem. Biophys. Res. Commun..

[63] Leenaars CHC (2019). Animal to human translation: A systematic scoping review of reported concordance rates. J. Transl. Med..

[64] Schram G, Pourrier M, Melnyk P, Nattel S (2002). Differential distribution of cardiac ion channel expression as a basis for regional specialization in electrical function. Circ. Res..

[65] Lavra L (2021). Generation and characterization of the human induced pluripotent stem cell (hiPSC) line NCUFi001-A from a patient carrying KCNQ1 G314S mutation. Stem Cell Res..

[66] Yang X (2014). Tri-iodo-l-thyronine promotes the maturation of human cardiomyocytes-derived from induced pluripotent stem cells. J. Mol. Cell Cardiol..

[67] Karakikes I, Ameen M, Termglinchan V, Wu JC (2015). Human induced pluripotent stem cell-derived cardiomyocytes: Insights into molecular, cellular, and functional phenotypes. Circ. Res..

[68] Ivashchenko CY (2013). Human-induced pluripotent stem cell-derived cardiomyocytes exhibit temporal changes in phenotype. Am. J. Physiol. Heart Circ. Physiol..

[69] Kumar N (2019). Assessment of temporal functional changes and miRNA profiling of human iPSC-derived cardiomyocytes. Sci. Rep..

[70] Minakhina S (2020). A direct comparison of thyroid hormone receptor protein levels in mice provides unexpected insights into thyroid hormone action. Thyroid.

[71] Anyetei-Anum CS, Roggero VR, Allison LA (2018). Localization of thyroid hormone receptor in target tissues. J. Endocrinol..

[72] George AL (2013). Molecular and genetic basis of sudden cardiac death. J. Clin. Investig..

[73] Němec J, Kim JJ, Salama G (2016). The link between abnormal calcium handling and electrical instability in acquired long QT syndrome—Does calcium precipitate arrhythmic storms?. Prog. Biophys. Mol. Biol..

[74] Abriel H, Rougier JS, Jalife J (2015). Ion channel macromolecular complexes in cardiomyocytes: Roles in sudden cardiac death. Circ. Res..

[75] Forini F, Nicolini G, Pitto L, Iervasi G (2019). Novel insight into the epigenetic and post-transcriptional control of cardiac gene expression by thyroid hormone. Front. Endocrinol..

[76] Blandin CE, Gravez BJ, Hatem SN, Balse E (2021). Remodeling of ion channel trafficking and cardiac arrhythmias. Cells.

[77] Dennis A, Wang L, Wan X, Ficker E (2007). hERG channel trafficking: Novel targets in drug-induced long QT syndrome. Biochem. Soc. Trans..

[78] Kuryshev YA (2005). Pentamidine-induced long QT syndrome and block of hERG trafficking. J. Pharmacol. Exp. Ther..

[79] Guilbot S, Morton M, Printemps R, Le Grand M (2020). Evaluation of two hERG channel trafficking modulators, pentamidine and pilsicainide, on stably transfected cell line and human induced pluripotent stem cell-derived cardiomyocytes. J. Pharmacol. Toxicol. Methods..

[80] Kitaguchi T (2016). CSAHi study: Evaluation of multi-electrode array in combination with human iPS cell-derived cardiomyocytes to predict drug-induced QT prolongation and arrhythmia—Effects of 7 reference compounds at 10 facilities. J. Pharmacol. Toxicol. Methods..

