# Supplementary material for: Thyroid hormones regulate cardiac repolarization and QT-interval related gene expression in hiPSC cardiomyocytes
Source: Sci Rep. 2022 Jan 12;12:568. doi: 10.1038/s41598-021-04659-w (PMC8755773; doi:10.1038/s41598-021-04659-w)
Supplement: Supplementary file 1 — Supplementary Figures. [file 41598_2021_4659_MOESM1_ESM.pdf]

Supplementary Figure 1

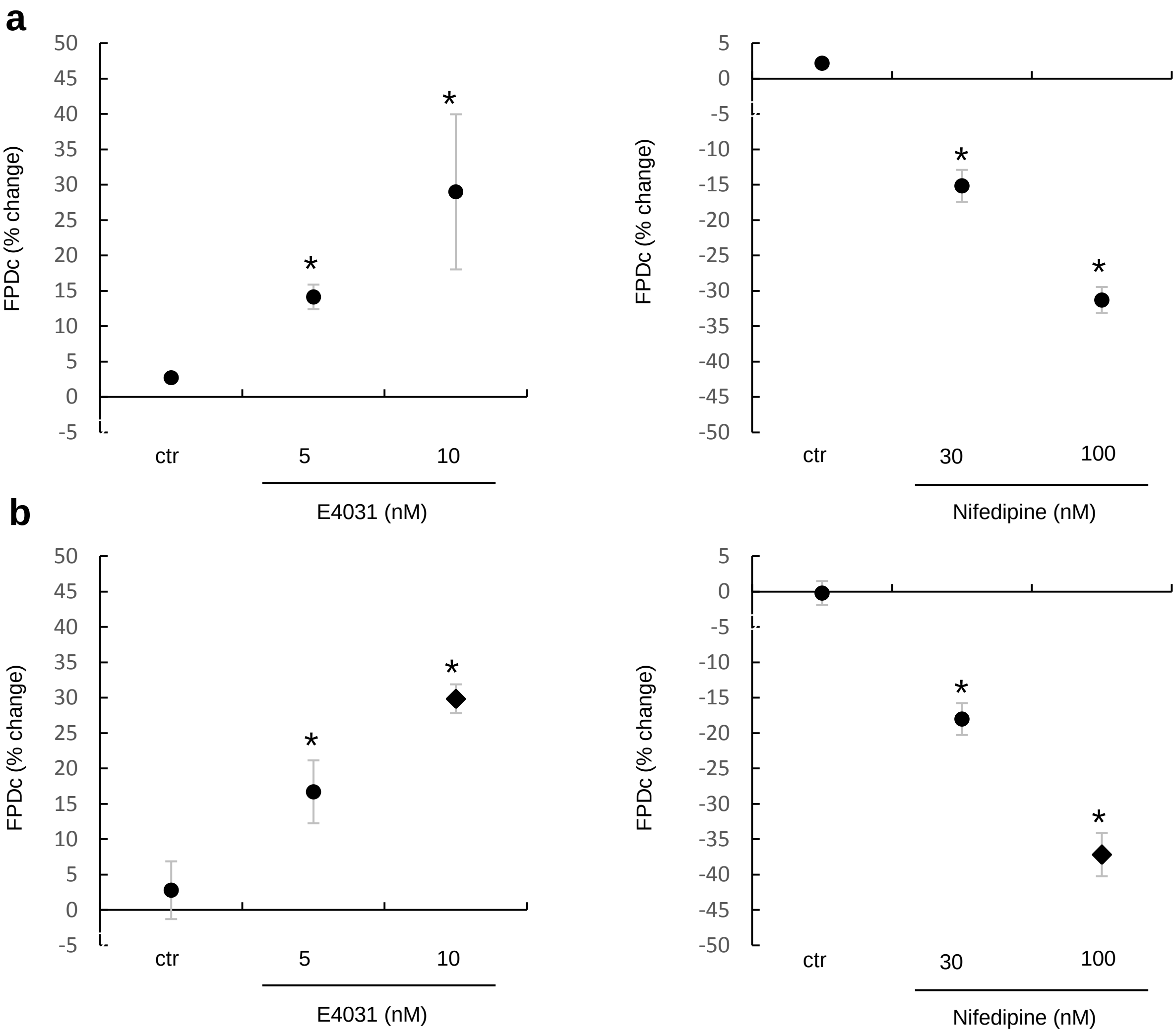

Supplementary Figure 1. Effect of E-4031 and Nifedipine treatments on FPD in hiPSC-CMs.

Analysis of FPDc in hiPSC-CMs treated for 30 min. with E-4031 (left panel) or Nifedipine (right panel) at the indicated doses, in iCM medium **(a)** or BMCC serum free media **(b)**; data are expressed as the percentage change of FPDc compared to baseline values. *$p<0.05$.

Supplementary Figure 2

a

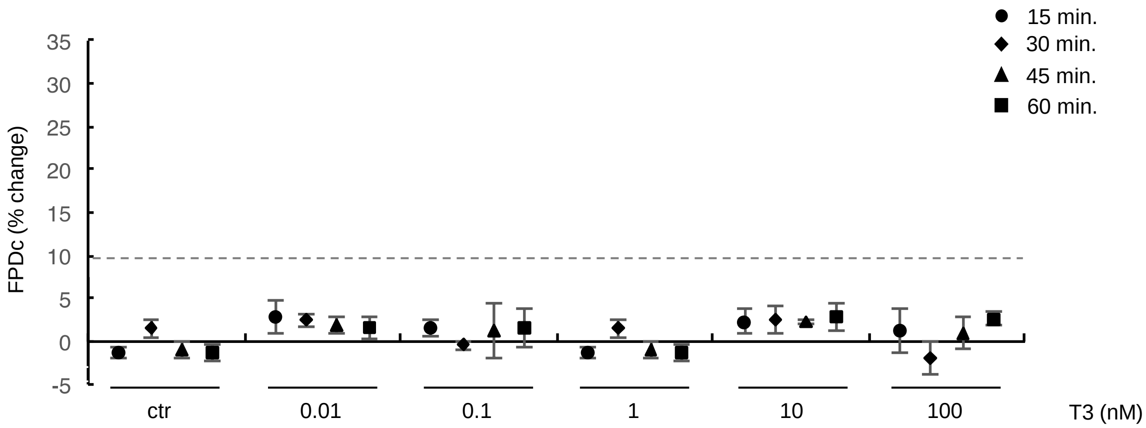

b

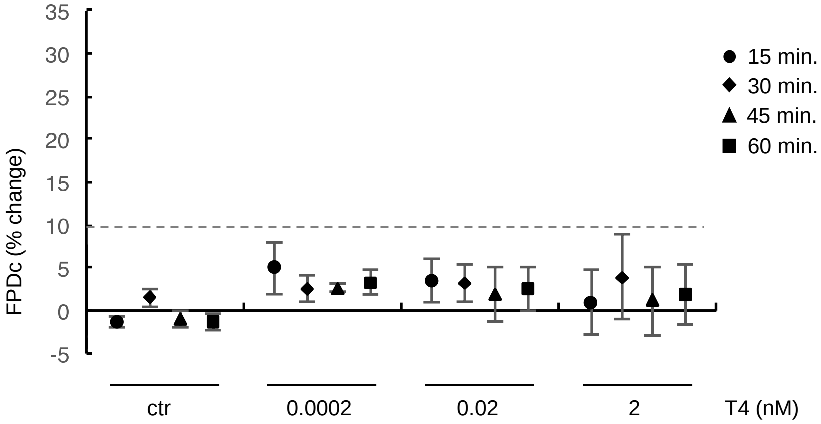

Short time effects of THs on FPDc in hiPSC-CMs.

a) Analysis of FPDc in hiPSC-CMs treated for 15 min, 30 min, 45 min and 60 min with T3 at different doses (0.01 nM, 0.1 nM, 1 nM, 10 nM and 100 nM)) compared with vehicle treated cells (ctr); data are expressed as the percentage change of FPDc compared to baseline values. **b)** Analysis of FPD in hiPSC-CMs treated for 15 min, 30 min, 45 min, 60 min with T4 at different doses (0.0002 nM , 0.02 nM , 2 nM), compared with vehicle treated cells (ctr); data are expressed as the percentage change of FPDc compared to baseline values.

Supplementary Figure 3

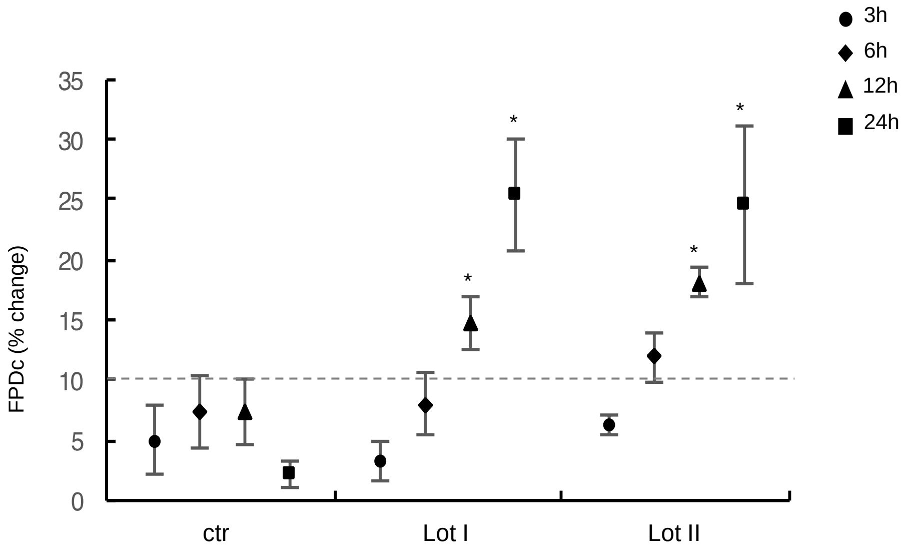

Supplementary Figure 3. Comparison of T3 effect on FPDc in two lots of the same hiPSC-CM cell line.

Analysis of FPDc in hiPSC-CMs of two different batches treated for 3, 6, 12 and 24 hrs with T3 1 nM, compared with EtOH (0.001%) treated cells (ctr); data are expressed as the percentage change of FPDc compared to baseline values. *p<0.01.

Supplementary Figure 4

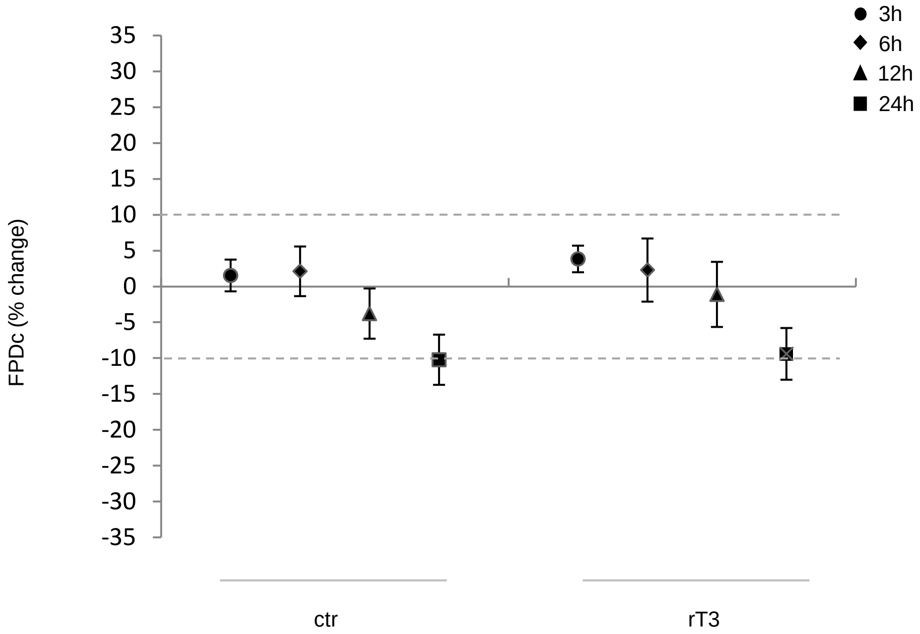

Supplementary Figure 4. Analysis of rT3 effect on FPDc in hiPSC-CMs.
Analysis of FPDc in hiPSC-CMs treated for 3, 6, 12 and 24 hrs with rT3 1 nM, compared with ammonia solution (0.01%) treated cells (ctr); data are expressed as the percentage change of FPDc compared to baseline values.

Supplementary Figure 5

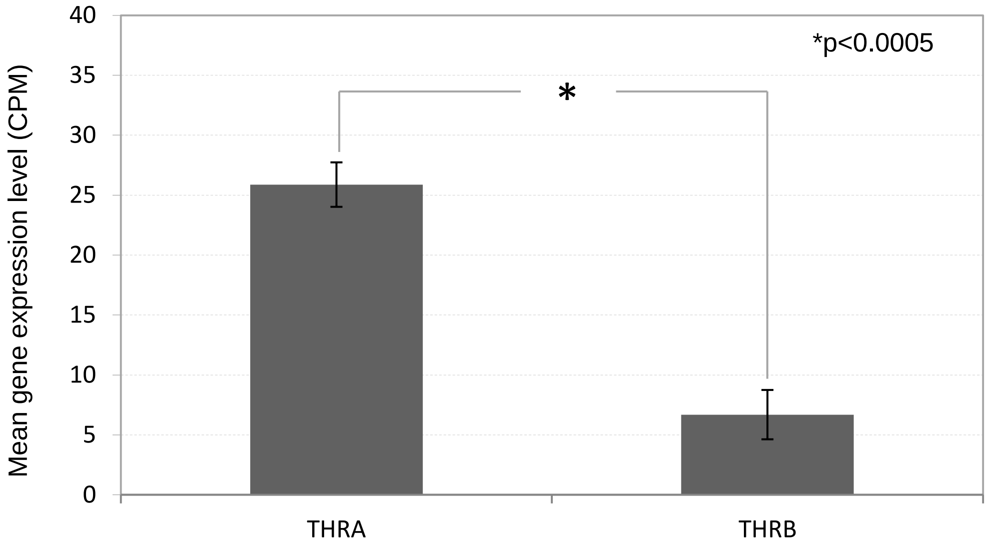

Supplementary Figure 5. THRA and THRB expression levels in hiPSC-CMs.
Analysis of mean expression levels of THRA and THRB in vehicle control iPSC-CMs. Data are extrapolated from RNAseq analysis data (GSE172348_Filtered_Normalized_Data_AvsB.xlsx).
Gene expression level is expressed as count form million (CPM). *p<0.0005
